# Complex problem solving—single ability or complex phenomenon?

**DOI:** 10.3389/fpsyg.2015.01669

**Published:** 2015-11-05

**Authors:** Wolfgang Schoppek, Andreas Fischer

**Affiliations:** ^1^Department of Education, Bayreuth UniversityBayreuth, Germany; ^2^Institute of Psychology, Heidelberg UniversityHeidelberg, Germany

**Keywords:** complex problem solving, individual differences, content validity, dynamic decision making

In recent years, large scale assessments such as PISA (OECD, 2014) have revived the interest in complex problem solving (CPS). In accordance with the constraints of such assessments, the focus was narrowed to psychometric aspects of the concept, and the minimal complex systems test MicroDYN has been propagated as an efficient instrument for measuring individual differences in CPS (Wüstenberg et al., 2012; Greiff et al., 2013). At present, MicroDYN is the most common psychometric instrument claiming to measure CPS^1^. MicroDYN consists of a number of linear systems with mostly three input and three output variables. The subjects have to explore each system, enter their insights into a causal diagram (representation phase) and subsequently steer the system to a given array of target values by entering input values (solution phase). Each system is attended to for about 5 min. MicroDYN yields reliable measures.

However, the validity of the approach is controversial. Funke (2014) has cautiously argued against the dominance of MicroDYN in the field of CPS. He pointed out that causal cognition plays a different role in the control of minimal complex systems than in the control of more complex and naturalistic systems. Therefore, according to Funke, CPS should not only be studied using minimal complex systems, but also with more realistic and complex simulated microworlds such as the Tailorshop (Danner et al., 2011a). Greiff and Martin (2014) countered that research with these microworlds has not succeeded in identifying “CPS as an unobserved latent attribute (i.e., a psychological concept)” (p.1).

The dispute between Funke (2014) and Greiff and Martin (2014) reflects the conflict between process-oriented vs. psychometric approaches to CPS^2^. In the present paper, we follow up on these two articles and add the notion that construing CPS as a single latent attribute might be unwarranted regarding the original and prevalent theoretical conception. In fact, originally, MicroDYN had not been constructed to measure CPS *per se*; rather it was devised as a “psychometric sound realization of selected but important CPS aspects” (Funke, 2010, p.138). Speaking of CPS as a latent attribute also obscures findings that MicroDYN assesses two discriminable—yet highly correlated—of these aspects (Wüstenberg et al., 2012; Greiff and Fischer, 2013; Fischer et al., 2015): knowledge acquisition, and knowledge application. Even though these aspects can be reliably assessed by MicroDYN they are not representative for CPS in general and there is more to CPS than MicroDYN does address (Fischer, 2015). In our opinion, “complex problem solving” is a multifaceted cognitive activity (Funke, 2014) rather than a single “latent attribute” (Greiff and Martin, 2014, p.1). We will present a number of arguments supporting this position and make suggestions for the further development of the field.

(1) Our first argument is based on the definition of CPS and the characteristic features of complex problems typically used in research: There is a problem whenever an organism wants to reach a goal but does not know how to do so (Duncker and Lees, 1945); and a problem is complex if a large number of highly interrelated aspects have to be considered in parallel (Dörner, 1997; Fischer et al., 2012). Problem solving is the activity of searching for a solution. The range of complex problems is heterogeneous and many researchers have noted that it is hard to define exhaustively the common features of the problems used (e.g., setting up a mobile phone or managing a corporation). In spite of this heterogeneity there is a list of five characteristic features that is uncontroversial among researchers in the field (Frensch and Funke, 1995; Fischer et al., 2012): A complex problem involves a relatively large number of interrelated variables (*complexity*). Therefore, each intervention to the complex problem has multiple consequences and there may be far- and side-effects (*interrelatedness*). The variables influence each other in ways that are not completely transparent to the problem solver (*intransparency*). The state of a complex problem is dynamically changing over time—as a result of the problem solver's actions or independent of them (*dynamics*). Last but not least, in CPS there usually are multiple, often contradictory goals to pursue (*polytely*). The processes for coping with these characteristics are heterogeneous, too: For example, identifying the most important variables in a system is different from analysing input-output sequences for underlying causal relations, which in turn is different from deriving adequate actions based on the problem's current state and the problem solver's assumptions about the underlying causal structure (cf. Fischer, 2015). Of course, these cognitive processes are expected to share variance in the efficiency of their execution commonly referred to as the g-factor. Whether these processes are correlated beyond g (indicating a one-dimensional CPS ability) is still an open question. There is some evidence that this is true for the facets of knowledge acquisition and knowledge application in MicroDYN (Sonnleitner et al., 2013). For other processes, this has not yet been shown and it is conceivable that low correlations among different microworlds are not always due to the low reliability of performance measures, but also to differences in how the microworlds call for different processes: Whereas the Tailorshop requires reduction of an overwhelming amount of information and planning, MicroDYN does much less so. Instead, solving MicroDYN items focuses more on interactive hypothesis testing (Fischer et al., 2015). So given these considerations, it does not seem reasonable to devise a single valid operationalization of CPS, nor to construe it as a one-dimensional ability construct. On the other hand, the attempt to assess important aspects of CPS quantitatively appears reasonable. From our point of view, this dilemma can be solved by combining different aspects of CPS performance into a competency (Fischer, 2015): A competency can be viewed as a formative construct—encompassing a set of knowledge elements and skills that need not necessarily be correlated (Jarvis et al., 2003). A person who is able and willing to solve a wide range of complex problems is competent in this regard. This argument is related to the content validity of the CPS construct, and therefore not backed up with empirical evidence. The predictive validity of a formative CPS competency is still to be established.

(2) A closer look at MicroDYN shows that it meets only a few of the criteria of complex problems. As the typical MicroDYN simulation involves only up to three input variables and the same number of dependent variables, it can be characterized as moderately complex. Also, interrelatedness of variables is mostly restricted to direct effects of independent variables: most simulations involve no effects among the dependent variables (side-effects). As a result, there are no conflicting goals—highly characteristic for polytelic situations—in current instances of the MicroDYN approach. Similarly, although MicroDYN simulations *could* include eigendynamics, most of them don't. In Greiff and Funke (2010), *no* items with eigendynamics were used; in Greiff et al. (2012) 64% of the Items in Study 1 and 42% of the Items in Study 2 involved eigendynamics. Moreover, eigendynamics cannot unfold their full effects due to the short input sequences of only four time steps in the solution phase. Furthermore, as the causal structure is revealed in the solution phase, intransparency is only given in the representation phase. An overview of how the two most common microworlds meet the five criteria is given in Table S1 (see Supplementary Material). From our point of view, these are severe shortcomings, because intransparency, conflicting goals, side effects, feedback loops, and eigendynamics are highly characteristic aspects of complex problems that require additional or different skills (Fischer et al., 2015) than those required by MicroDYN. To investigate how humans handle dynamic systems, longer simulations should be used—preferably real-time driven (Schoppek and Fischer, 2014).

(3) Empirically, MicroDYN performance shares much variance with measures of fluid intelligence (Wüstenberg et al., 2012; Greiff and Fischer, 2013; Greiff et al., 2013). This is exactly what one would expect of a reliable and valid measure of CPS competency (Fischer et al., 2015). To justify the assumption of a new psychometric construct, it must be shown that it can account for incremental variance in adequate criteria. This has been tried repeatedly, predominantly using school grades. The modal result of these studies is that MicroDYN-CPS accounts for 5% variance in school grades incremental to fluid intelligence. We challenge the value of school grades as primary criterion for validating MicroDYN for two reasons: In contrast to MicroDYN, they are highly aggregated measures. This violates Brunswik symmetry, which requires that concepts be located on similar levels of aggregation in order to validate each other (Wittmann and Hattrup, 2004). Also, school grades are awarded based on a number of poorly specified and heterogeneous principles—problem solving being probably a minor one. Therefore, the range of criteria for validating MicroDYN scores should be extended. In particular, more complex and more dynamic operationalizations of CPS, such as “Tailorshop” or “Dynamis2” (Schoppek and Fischer, 2014) should be used for validation. Recent research has shown that performance data with good psychometric properties can be gathered with such microworlds (Wittmann and Hattrup, 2004; Danner et al., 2011a). Ideally, performance in MicroDYN accounts for incremental variance in other microworlds, whereas additional variance can be explained by other variables such as domain specific knowledge. That is to say we don't agree with Greiff and Martin (2014) that these variables reflect nothing but unsystematic variance. When such validation studies reveal that MicroDYN explains substantial unique variance in other complex systems, it can be used as an efficient screening test for CPS competency, even if it covers only a subset of the pertinent skills.

(4) Our last argument concerns the value of the psychometric approach for analyzing CPS. Again, this has to do with the question, if something multifaceted like CPS should be construed as ability construct. Investigating how a construct contributes to the prediction of interesting criteria is valuable, particularly in applied contexts. But it is neither sufficient nor even very helpful for identifying underlying processes. Let us clarify this issue by means of a thought experiment: Consider a model that simulates problem solving performance as a result of four processes. Assume each process depends on motivation and intelligence in the following way: The probability to begin the process is determined by motivation and the probability to accomplish the process successfully (given it has been begun) is determined by intelligence. The functional relationships between motivation, intelligence and the respective probabilities are modeled with logistic functions like those shown in Figure 1. The level of problem solving performance is represented by the number of successfully accomplished processes^3^. Intelligence and motivation are assumed to be normally distributed and uncorrelated. Based on a simple model like this one may be tempted to expect high correlations between problem solving performance and intelligence and motivation, respectively.

**Figure 1:**
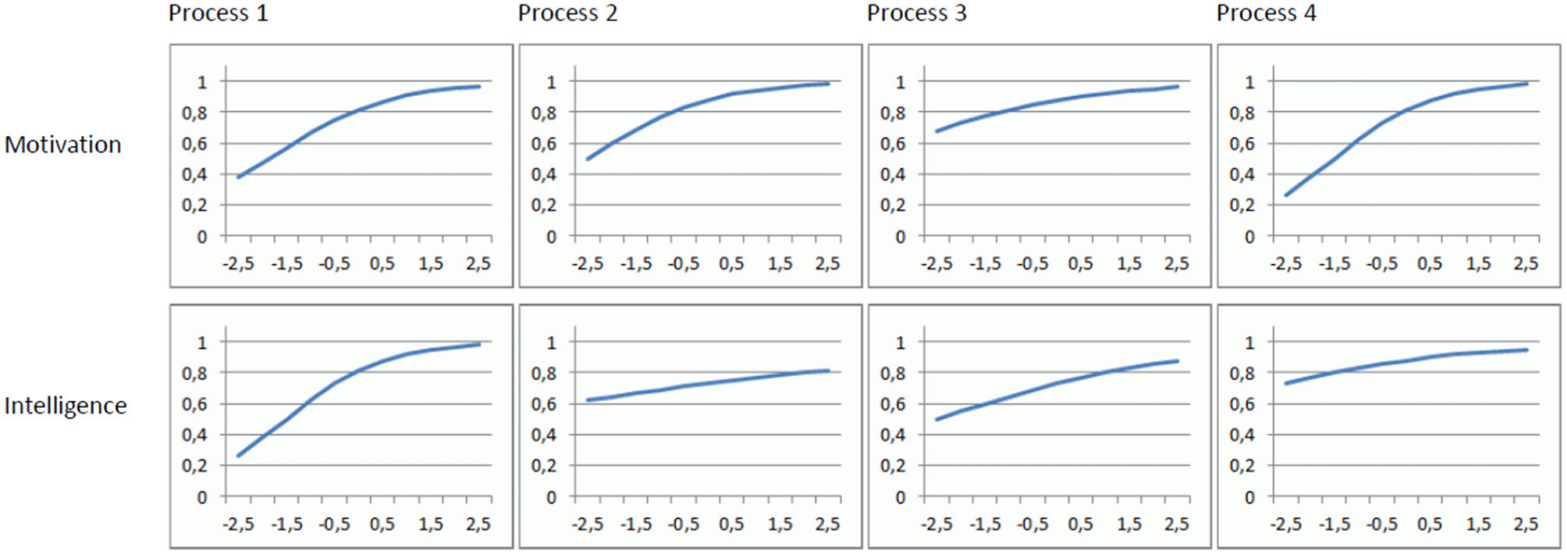
**Functional relations between the simulated individual differences variables “Motivation” and “Intelligence” and the probabilities of starting and accomplishing each process**. For example, with a motivation of 0.0 (interpreted as a *z*-value) the probability of starting Process 1 equals 0.8.

We have simulated *N* = 1000 samples of *n* = 500 cases using the functions shown in Figure 1, calculated a multiple regression analysis for each sample, and extracted the standardized regression weights and the *R*^2^.

The mean regression weights in this simulation were β_1_ = 0.321 (*SD* = 0.04) for motivation and β_2_ = 0.254 (*SD* = 0.04) for intelligence. The mean *R*^2^ was 0.170. Given that problem solving in our model solely depends on intelligence and motivation, these regression weights are remarkably low.

Two regression parameters and an *R*^2^-value cannot convey complete information about the eight functions that produced the results. Stated differently, many combinations of producing functions will lead to the same psychometric results. On the other hand, the thought experiment also shows that reliable assessment of CPS (or its various aspects) is necessary for validating process models. So we can conclude that the seemingly conflicting process-oriented vs. psychometric approaches depend on each other and therefore, should stimulate each other. However, to make progress in understanding CPS, we need process-oriented theories that make specific predictions. For example, it could be construed within a dual processing framework (Evans Jonathan, 2012) or the joint role of motivation and executive control in CPS could be investigated (Scholer et al., 2010).

As the main claim of this paper is that CPS should not be construed as a single ability construct, we want to end it with a preliminary list of exemplary constituents that should be included in a comprehensive measure of CPS competency^4^. Among the knowledge-related components of CPS competency we distinguish (1) knowledge of concepts related to CPS and (2) knowledge of strategies, tactics, and operations, and when to apply them:

Knowledge of concepts:

- Causal loops (e.g., vicious circle), predator-prey systems, exponential growth, saturation, etc.
- Delayed effects.
- Polytelic goal structures.

Knowledge of strategies/tactics/operations:

- Analysis of effects and dependencies in order to determine importance of variables as well as far- and side-effects (Dörner, 1997).
- Cross-impact analysis for identifying a variable's importance with respect to one's goals (Vester, 2007).
- Control of variables strategy aka VOTAT (vary one thing at a time).
- Applying input impulses and observing their propagation through the system to identify eigendynamics.
- Utility analysis for coping with politely.

MicroDYN is sensitive to the control of variables strategy and covers processes such as “interactively identifying causal relations between pairs of variables” or “deriving a plan from causal knowledge.” For these aspects it can be regarded as a useful, well established measure. At the same time it is obvious that there are other important components of CPS competency that cannot be assessed with MicroDYN (cf. Fischer et al., 2015)—e.g., skills for reducing complexity. To investigate and assess the set of skills, that is required to solve a wide range of complex problems, a greater variety of tests is necessary.

## Conflict of interest statement

The authors declare that the research was conducted in the absence of any commercial or financial relationships that could be construed as a potential conflict of interest.

## Footnotes

^1^For simplicity, we subsume the microworld GeneticsLab (Sonnleitner et al., 2013) under the MicroDYN approach because of its close similarity to MicroDYN.

^2^Please see Buchner (1995) for a detailed discussion of different approaches to CPS. The mentioned conflict has a long-standing equivalent in intelligence research, where information-processing approaches, which strive to discover the “mechanics of intelligence” (Hunt, 2011, p.140), compete with psychometric approaches.

^3^The number of processes is arbitrary and the exact nature of the processes does not matter for the point at hand. Please note that the results of the regression analyses depend on the parameters of the logistic functions: Functions steeper than those in Figure 1 result in higher *R*^2^s.

^4^Please note, there may be emotional and motivational constituents of CPS competency as well, e.g., frustration tolerance, But given the cognitive nature of our argument, they are beyond the scope of this paper.
