# Supplementary material for: Complex problem solving—single ability or complex phenomenon?
Source: Front Psychol. 2015 Nov 5;6:1669. doi: 10.3389/fpsyg.2015.01669 (PMC4633517; doi:10.3389/fpsyg.2015.01669)
Supplement: Supplementary file 1 [file Table1.PDF]

Table S1. Overview of how the two common microworlds MicroDYN and Tailorshop meet the five criteria for complex problem solving.

Criteria	*MicroDYN*	*Tailorshop*
**complexity**	low	high
**interrelatedness**	low in most instances	moderate
**intransparency**	given only in the knowledge acquisition phase	given
**dynamics**	very reduced, no eigendynamics in many items	given, eigendynamics are present
**polytely**	no contradicting goals	contradicting goals
**Psychometric reliability**	.74-.85	.52-.96

notes. Reliability estimates are taken from Wüstenberg, Greiff, & Funke (2012), Greiff et al., (2013), Danner et al. (2011a) and the first measurement occasion of Danner (2011b).
